# Propionic Acid Targets the TLR4/NF-*κ*B Signaling Pathway and Inhibits LPS-Induced Intestinal Barrier Dysfunction: *In Vitro* and *In Vivo* Studies

**DOI:** 10.3389/fphar.2020.573475

**Published:** 2020-09-18

**Authors:** Randong Yang, Xiaoxiao Hu, Xianzheng Xie, Haiqiong Chen, Huangyi Fang, Libing Zhu, Zhongrong Li

**Affiliations:** ^1^ Department of Pediatric Surgery, The Second Affiliated Hospital & Yuying Children’s Hospital of Wenzhou Medical University, Wenzhou, China; ^2^ Department of Pediatric Surgery, The First Affiliated Hospital of Wenzhou Medical University, Wenzhou, China

**Keywords:** propionic acid, lipopolysaccharide, intestinal barrier, cell migration, NLRP3 inflammasome

## Abstract

Intestinal barrier dysfunction contributes to the development of intestinal diseases. Propionic acid (PA), a metabolite generated by anaerobic fermentation of dietary fiber in the intestinal cavity, has been proved to exert anti-inflammatory effects in a variety of diseases. However, the exact role of PA in LPS-induced intestinal barrier dysfunction is still unclear. Accordingly, we examined the latent mechanism of PA and its protective role in LPS-induced intestinal barrier dysfunction by both *in vitro* and *in vivo* experiments. *In vitro*, we identified that PA treatment could strongly promote cell migration, inhibit activation of NLRP3 inflammasome and maintain intestinal barrier function in LPS-induced IEC-6 cells, indicating the protective effect on the intestinal barrier function of PA. Further investigation of the mechanism involved revealed that PA could suppress the activation of TLR4/NF-*κ*B pathway. *In vivo*, in a LPS-induced rat model, PA-induced protective effects in intestinal barrier dysfunction could be detected. In summary, our findings clarify the role of PA in intestinal barrier dysfunction and suggest that it is promising for the treatment of LPS-related intestinal diseases.

## Introduction

As one of the most important defense barriers of the body, an intact intestinal barrier is a physical and functional barrier formed by intestinal epithelial cells, and their intercellular connections can regulate the absorption of nutrients and defend against the translocation of toxins, antigens, and enteric pathogenic organisms (Turner, 2006). Intestinal barrier dysfunction can lead to increased intestinal permeability of lumen endotoxin and a significant pro-inflammatory state in the intestine (Gil-Cardoso et al., 2019). The dysfunction of intestinal barrier is an indispensable characteristic in a variety of intestinal diseases such as necrotizing enterocolitis (NEC) and inflammatory bowel disease (IBD) (Salim and Soderholm, 2011; Halpern and Denning, 2015). Thus, dysfunction of intestinal barrier imparts a significant and negative impact on human health; consequently an understanding of the underlying mechanisms involved is of great significance in the development of effective prevention and treatment strategies.

It is well known that intestinal epithelial cells and their intercellular structural integrity are essential for the maintenance of intestinal barrier function (Kucharzik et al., 2001). Tight junctions (TJs), as the most apical intercellular junctions of epithelial cells, play a vital role in the regulation of the intercellular structural integrity (Takuya, 2013). Lipopolysaccharide (LPS) has been proved to participate in the destruction of intestinal barrier (Gil-Cardoso et al., 2019). It has been documented that LPS can decrease the synthesis of TJs, thereby improving intestinal epithelial permeability and resulting in intestinal barrier dysfunction (Bein et al., 2017). What’s more, LPS cannot only inhibit cell migration, but also activate NLRP3 inflammasome in intestinal epithelial cells (Reed et al., 2019). Activation of NLRP3 inflammasome is considered to be a key step in detecting cellular damage and mediating inflammatory responses. NLRP3 inflammasome induces the secretion of the mature form of proinflammatory cytokines interleukin IL-1*β* and IL-18 through casepase-1 activation, which plays an important role in inflammatory diseases (Rahim et al., 2017). Meanwhile, cell migration and NLRP3 inflammasome have been shown to be inextricably linked to intestinal barrier function. Either decreased cell migration or over-activation of NLRP3 inflammasome can induce intestinal barrier dysfunction (Lei-Leston et al., 2017; Reed et al., 2019). Therefore, improving cell migration and inhibiting activation of NLRP3 inflammasome in intestinal epithelial cells, theoretically, can contribute to the restoration of the intestinal barrier function.

Dietary fiber is an important component of enteral nutrition, which is anaerobic fermented into Short-chain fatty acids (SCFAs) in the intestinal cavity (Bolognini et al., 2016). The intestinal SCFA concentration can range from 60 to 150 mmol/L, with butyric, propionic, and acetic acid in a nearly constant molar ratio of 15:25:60, respectively (Hill, 1995; D’Argenio and Mazzacca, 1999). Besides, SCFAs have recently attracted considerable interest because of their possible importance for host health. Additionally, the beneficial effects of SCFAs on various aspects of intestinal physiology and metabolism have been well documented (Al-Lahham et al., 2010). Most previous studies mainly focused on either solely on the role of butyric acid alone (Hamer et al., 2008) or on the effects of complex SCFA mixtures (Feng et al., 2018). However, few studies have devoted their efforts to other SCFAs such as propionic acid (PA), although it is abundant as butyric acid in the gut and blood. Furthermore, the role of PA in LPS-stimulated intestinal barrier has not been reported. In the current study, we attempted to investigate whether PA defends against intestinal barrier dysfunction assaulted by LPS and to further illuminate the mechanism underlying PA-exerted protection for intestinal barrier.

## Materials and Methods

### Cell Culture and Management

Intestinal epithelial IEC-6 cells (Cell Bank of the Chinese Academy of Sciences, China) were cultured in DMEM supplemented with 10% fetal bovine serum, 1% insulin, and 1% antibiotics (100 U/ml penicillin and 100 U/ml streptomycin) at 37°C with 5% CO_2_ in a humidified incubator.

All cells were washed with PBS before incubation with PA (St. Louis, MO) or Lipopolysaccharides (LPS, 055:B5, Life Sciences Co. Ltd., Beijing, China) in culture medium with FBS. After reaching 90% confluence, the cells were treated with media containing different concentrations of PA for 24 h with or without LPS. The following five groups of IEC-6 cells were established: an untreated control group, a group treated with medium supplemented with 10 μg/ml LPS, a group treated with medium supplemented with 0.5 mM PA and 10 μg/ml LPS, a group treated with medium supplemented with 5 mM PA and 10 μg/ml LPS, and a group treated with medium supplemented with 5 mM PA. Cell cultures were maintained following standard laboratory protocols.

### Experimental Animals and Management

Sixty male Sprague Dawley rats (from the Animal Center of Chinese Academy of Sciences, Shanghai, China), weighing 200 ± 20 g, were adapted to 25°C and 60% relative humidity, and maintained under a 12:12 h light:dark cycle with free access to water and food.

The rats were randomly assigned to four groups (n = 15 per group) including a control group, a LPS group, a LPS + PA group, and a PA group. The rats in the control and PA groups received 20 ml/kg of drinking water and 200 mM PA, respectively, for 14 consecutive days. The concentration and the method of administration of LPS were referred to Yin et al. (Yin et al., 2019). The rats in the LPS group were injected LPS (10 mg/kg) intraperitoneally on day 13. The rats in the LPS + PA group received 20 ml/kg of 200 mM PA for 14 days and were injected LPS (10 mg/kg) intraperitoneally on day 13. The rats were killed 24 h later. The ileal intestine was collected and immediately flushed with saline to remove all intestinal contents.

### Cell Viability Assay

Cell viability was evaluated by an MTT assay according to the methods described by Song et al. (2016), with some modifications. In brief, IEC-6 cells were pre-incubated in 96-well plates. After incubation with different concentrations of PA and LPS for 24 h, the treated cells in each well were supplemented with 100 μl of culture medium containing 0.5 mg/ml MTT. The plates were incubated for an additional 4 h at 37°C. The MTT medium was discarded and 200 μl of dimethyl sulfoxide was added to dissolve formazan. Absorbance was monitored at 570 nm using a Microplate Reader (Thermo, Waltham, MA). Cell viability was calculated as the optical density for each treatment group relative to the optical density for the control group (expressed as a percentage).

### Transepithelial Electrical Resistance Measurements

The permeability of IEC-6 monolayers was detected by TEER measurement (Zhuang et al., 2019). The control, LPS, 0.5 mM PA + LPS, 5 mM PA + LPS, and 5 mM PA IEC-6 cells were seeded on 24-well Transwell filters and were grown as monolayers for 6 days. Subsequently, we added the determined PA and LPS for 24 h. The resistance value of each well was measured by an epithelial voltohmmeter ERS-2 (Merck Millipore, USA).

### Migration Assay

A migration assay was performed mainly as reported previously (Hoying and Williams, 1996). Cells cultured to confluence in six-well plates were starved for 12 h. The cell monolayer was scratched by a standard 200-μl pipette tip across the diameter of the wells. Cells were treated with LPS with or without PA according to the experimental design. After incubation, images were obtained using a digital camera, and cell areas were measured using ImageJ version 2.1 (Bethesda, MD). The migration area in each wound was calculated using the following formula: cell-free area at a fixed time/cell free area at 0 h. If necessary, contrast and brightness were digitally optimized.

### Western Blot Analysis

Western blot analysis was evaluated according to the methods described by Elamin et al. (2013). The proteins of IEC-6 cells and ileum tissues were lysed in RIPA buffer (ThermoFisher Scientific) containing 1% protease inhibitor cocktail. Total proteins in the supernatants were collected by centrifugation, and the protein concentration was measured using the Enhanced BCA Protein Assay Kit (Thermo, Waltham, MA). Proteins were separated by SDS-PAGE and transferred onto PVDF membranes. After blocking for nonspecific binding, the membranes were incubated overnight at 4°C with primary antibodies. The membranes were incubated with secondary antibody for 2 h at room temperature. Immunocomplexes were visualized using an Enhanced Chemiluminescence (ECL) Kit according to the manufacturer’s instructions (Thermo, Waltham, MA). Expression levels were normalized to GAPDH levels and analyzed using ImageJ.

### Immunofluorescence Assay

Immunofluorescence assay was evaluated according to the methods described by Zhuang et al. (2019). Cells cultured on a coverslip were fixed with 4% paraformaldehyde. After they were permeabilized with 0.1% Triton X-100, the cells were blocked in BSA. Then, the cells were incubated with primary antibodies in a humid chamber overnight at 4°C. Cells were then incubated with Alexa Fluor 488-labeled or Alexa Fluor 594-conjugated secondary antibodies. Cells were then incubated with DAPI and sealed with a coverslip. Images were captured using a fluorescence microscope (Olympus Inc., Tokyo, Japan). Fluorescence intensity was measured using ImageJ.

### Histopathological Examination

Histopathological examination was evaluated according to the methods described by Tong et al. (2016). The ileum tissue (three per group) collected from rats in different groups were excised and fixed in 4% paraformaldehyde. They were then embedded in paraffin, cut, and stained with the hematoxylin and eosin (HE) reagent. The images were observed using a light microscope (×100 and ×200 magnification).

### Immunohistochemistry

Immunohistochemistry was evaluated according to the methods described by Yin et al. (2019). The expression of tight junction proteins of ileal tissues was assessed by immunohistochemistry. The sections embedded in paraffin were deparaffinized and rehydrated and underwent heat pretreatment for antigen retrieval. After quenching endogenous peroxidase and blocking non-specific bindings, sections were incubated with 1:100 diluted specific antibodies and finally incubated with HRP-conjugated secondary antibody. The images were observed using a light microscope (×200 and ×400 magnification). Three fields were randomly selected and used for quantitative analysis.

### In Vivo Intestinal Permeability

Intestinal permeability was evaluated according to the methods described by Guo et al. (2019). The rats were gavage fed with 10 mg/ml FITC-dextran (FD10S; Thermo, Waltham, MA) in a total of 40 mg/100g body weight. After 5 h, blood was collected by terminal cardiac puncture and centrifuged at 3,000g for 10 min. Synergy H4+ (BioTek) was used to detect the fluorescence intensity with 485 nm excitation light and 530 nm absorption light. The serum concentration of FD10S was quantified using a fluorimeter (SpectraMax M2e, Tokyo, Japan).

### Statistical Analysis

Data are presented as averages ± standard deviation. The statistical significance of differences among groups was evaluated by one-way analysis of variance (ANOVA) and Tukey’s test for multiple comparisons. Statistical analyses were performed using Graph Pad Prism 6. Differences were considered statically significant at P < 0.05. All experiments were repeated at least three times to ensure reproducibility.

## Results

### Selection of Appropriate LPS and PA Concentrations for IEC-6 Cell Viability

**Figure 1:**
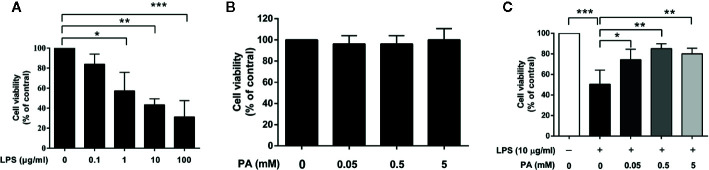
The effects of LPS and PA on IEC-6 cells’ viability. Cell viabilities were assayed by the MTT method. **(A)** IEC-6 cells were stimulated with different concentrations of LPS (0, 0.1, 1, 10, and 100 μg/ml) for 24 h. **(B)** IEC-6 cells were treated with PA at a series of concentrations (0, 0.05, 0.5, and 5 mM) for 24 h. **(C)** IEC-6 cells were treated with PA (0, 0.05, 0.5, and 5 mM) and LPS (l0 μg/ml) for 24 h. The data represent the averages ± S.D. Significant differences between the treatment and control groups are indicated as *P < 0.05, **P < 0.01, ***P < 0.001. Significance was determined by one-way ANOVA.

To determine the suitable concentrations of LPS and PA for subsequent experiments, cell viability was monitored by MTT assays. IEC-6 cells were incubated with different concentrations of LPS (0, 0.1, 1, 10, and 100 μg/ml) and PA (0, 0.05, 0.5, and 5 mM) for 24 h. As shown in **Figure 1A**, compared with the control group, LPS provoked a significant decrease in cell viability in a concentration-dependent manner. In general, the concentration of a compound resulting in approximately 50% cell viability is considered suitable for studies of cytotoxicity. In this study, exposure to LPS at 10 μg/ml resulted in a 50% reduction in cell viability and was selected for subsequent analysis. As shown in **Figure 1B**, cell viability was not significantly affected by PA, suggesting that PA was non-toxic at these concentrations and could be used for further investigation.

### PA Recovers Cell Viability in LPS-Induced IEC-6 Cells

To evaluate the protective effect of PA on LPS-induced cell toxicity, IEC-6 cells were treated with PA (0, 0.05, 0.5, and 5 mM) and LPS (10 μg/ml) for 24 h. As summarized in **Figure 1C**, PA treatment at these concentrations attenuated the inhibitory effect of LPS on cell viability. A concentration of 0.5 mM PA resulted in the greatest attenuation of LPS-induced cell toxicity (P < 0.01). Therefore, PA can enhance the viability of LPS-induced IEC-6 cells.

### PA Alleviates Intestinal Barrier Dysfunction in LPS-Induced IEC-6 Cells

**Figure 2:**
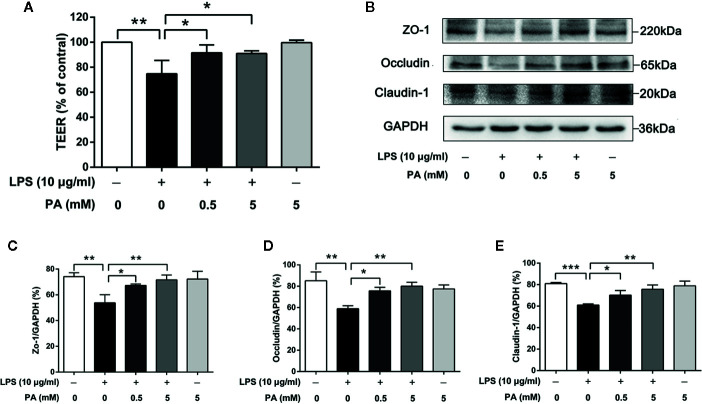
The effects of PA on TEER and expression of tight junction proteins in LPS-induced IEC-6 cells. Cells were cultured in media with varying concentrations of PA (0, 0.5 and 5 mM) and LPS (l0 μg/ml) for 24 h. The indicated PA concentrations were maintained throughout the duration of the experiment. TEER was measured by epithelial voltohmmeter ERS-2. **(A)** The value of TEER. **(B)** The protein expressions of ZO-1, occludin, claudin-1 were determined by western blot analysis and were quantified in **(C–E)**. The data in the figures represent the averages ± S.D. Significant differences between the treatment and control groups are indicated as *P < 0.05, **P < 0.01, ***P < 0.001. Significance was determined by one-way ANOVA.

In order to determine the effect of PA on the intestinal barrier function in LPS-induced IEC-6 cells, we measured TEER over 24 h followed by PA exposure. As shown in **Figure 2A**, LPS significantly decreased the level of TEER (P < 0.01), while treatment with PA moderated the reduced TEER (P < 0.05). Secondly, we utilized immunoblotting to analyze the protein expression levels of ZO-1, occludin, and claudin-1. As shown in **Figures 2B–E**, the protein expression levels of ZO-1, occludin, and claudin-1 were lower in the LPS group than in the control group (P < 0.01). This LPS-induced alteration of tight junction protein expression was inhibited by PA (P < 0.05). These results suggest that PA can enhance and protect intestinal barrier function against LPS in IEC-6 cells.

### PA Promotes Epithelial Cell Migration *via* Inhibition of Expression of β1-Integrin in LPS-Induced IEC-6 Cells

**Figure 3:**
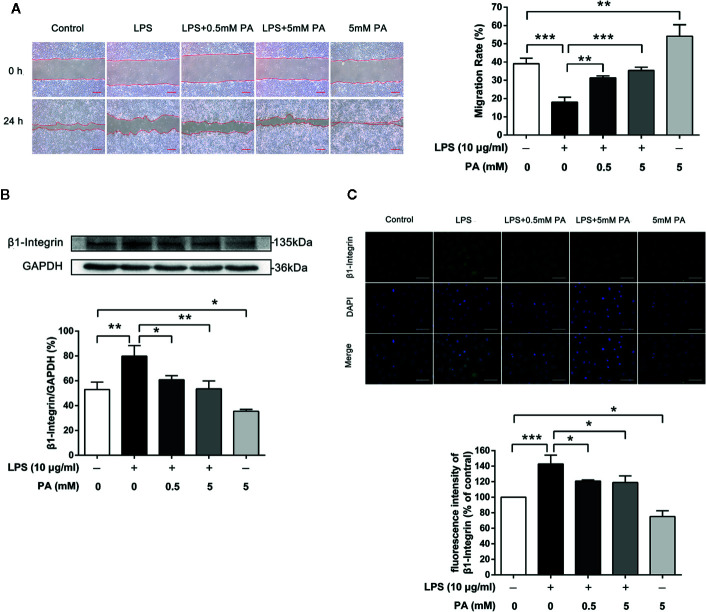
The effects of PA on cell migration and expression of *β*1-integrin protein in LPS-induced IEC-6 cells. IEC-6 cells were scrape-wounded and exposed to 0, 0.5, or 5 mM PA with or without LPS as described in *Materials and Methods*. **(A)** Images of cellular wounds generated were photographed at 0 and 24 h (scale bar: 0.3 mm). Wound healing of IEC-6 cells was assessed by comparing the wound area at 24 h after treatment with that prior to treatment. **(B)** The expression of β1-integrin was examined by western blot. **(C)** The expression of β*1*-integrin was detected by immunofluorescence combined with DAPI staining for nuclei (scale bar: 50 μm). The fluorescence intensities of NLRP3 and caspase-1 were determined using Image J software. The data in the figures represent the averages ± S.D. Significant differences between the treatment and control groups are indicated as *P < 0.05, **P < 0.01, ***P < 0.001. Significance was determined by one-way ANOVA.

We explored the effect of PA on cell migration under inflammatory conditions. Intestinal epithelial cell restitution in a well-established wounding model of confluent monolayers of IEC-6 cells was tested after treatment with PA or LPS for 24 h. As shown in **Figure 3A**, LPS notably inhibited wound healing of IEC-6 cells after treatment with LPS (P < 0.001), while treatment with PA had notable effect on wound healing (P < 0.01). Meanwhile, PA (5 mM) resulted in greater wound repair in IEC-6 cell monolayers than that in the control group (P < 0.01). PA facilitates IEC-6 migration, which requires the dynamic turnover of cell–matrix associations. Accordingly, we investigated the effect of PA on the adhesion of epithelial cells to a complex extracellular matrix. Our results showed that PA could reverse the LPS-mediated increase in *β*1-integrin protein expression (P < 0.05; **Figure 3B**). Finally, we used immunofluorescence assay to analyze the protein expression of *β*1-integrin. As illustrated in **Figure 3C**, the protein expression of *β*1-integrin was decreased by PA (P < 0.05). These data indicate that PA promotes epithelial cell migration *via* inhibition of expression of *β*1-integrin induced by LPS.

### PA Inhibits the Activation of NLRP3 Inflammasome in LPS-Induced IEC-6 Cells

**Figure 4:**
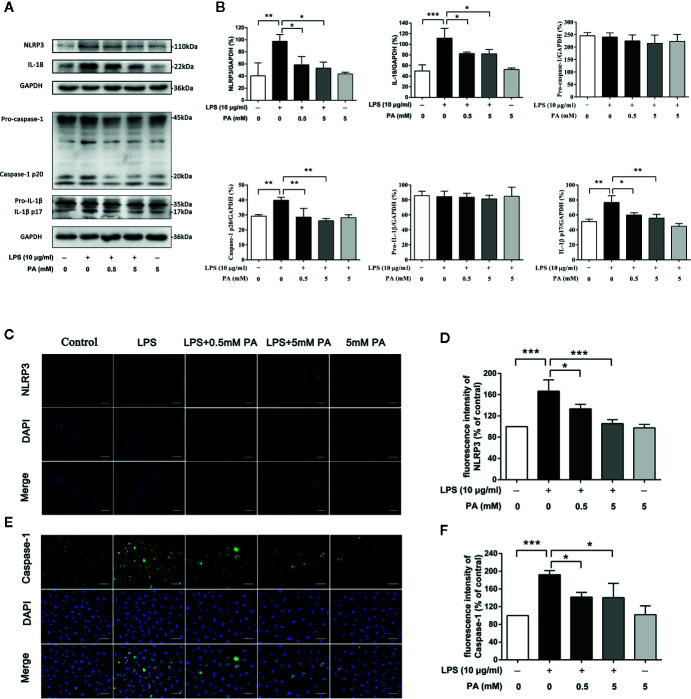
The effects of PA on the activation of NLRP3 inflammasome in LPS-treated IEC-6 cells. **(A)** The expressions of NLRP3, pro-caspase-1, caspase-1, pro-IL-1*β*, IL-1*β*, and IL-18 at protein levels were determined by western blot analysis and were quantified in **(B)**. **(C)** NLRP3 and **(E)** caspase-1 were detected by immunofluorescence combined with DAPI staining for nuclei (scale bar: 30 μm). The fluorescence intensities of **(D)** NLRP3 and **(F)** caspase-1 were determined using Image J software. The data represent the averages ± S.D. Significant differences between the treatment and control groups are indicated as *P < 0.05, **P < 0.01, ***P < 0.001. Significance was determined by one-way ANOVA.

To explore whether PA inhibits the activation of NLRP3 inflammasome, we measured levels of inflammasome markers, NLRP3, caspase-1, IL-1*β*, and IL-18. As indicated in **Figures 4A, B**, treatment with PA rescued the obvious increases in protein expression in NLRP3, caspase-1, IL-1*β*, and IL-18 induced by LPS. Meanwhile, it was also found that PA had no effect on the precursors of caspase-1 and IL-1*β*. To confirm these results, immunofluorescence was used to detect the localization and expression of NLRP3 (red) and caspase-1 (green) for a more intuitive analysis. As shown in **Figures 4C–F**, treatment with PA reduced the expression of NLRP3 and caspase-1. These results were in accordance with western blotting results. These findings suggest that the LPS-induced activation of NLRP3 inflammasome is inhibited by PA.

### PA Activates TLR4/NF-κB Pathway in LPS-Induced IEC-6 Cells

**Figure 5:**
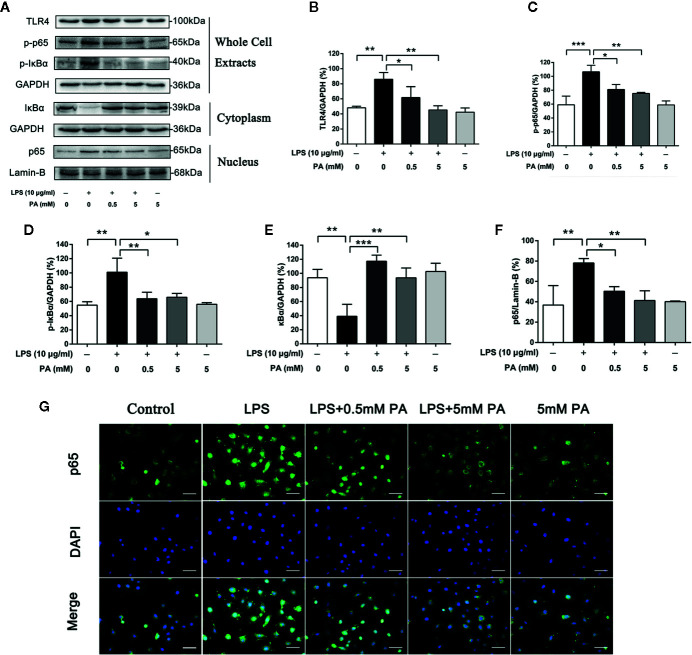
The Effects of PA on the activation of TLR4/NF-*κ*B pathway in LPS-induced IEC-6 cells. **(A)** The protein expressions of TLR4, p-I*κ*B*α* and p-p65 in whole cell extracts, I*κ*B*α* in the cytoplasm, and p65 in the nucleus in IEC-6 cells treated as above were visualized by western blot and were quantified in **(B–F)**. Relative expression of p-I*κ*B*α* in whole cell extracts, p-p65 in whole cell extracts, and I*κ*B*α* in the cytoplasm was expressed as a percentage of the level of GAPDH. Meanwhile, relative expression of p65 in the nucleus was expressed as a percentage of the level of Lamin-B. **(G)** The nuclear translocation of p65 was detected by immunofluorescence combined with DAPI staining for nuclei (scale bar: 30 μm). The data represent the averages ± S.D. Significant differences between the treatment and control groups are indicated as *P < 0.05, **P < 0.01, ***P < 0.001. Significance was determined by one-way ANOVA.

TLR4/NF-*κ*B pathway plays a crucial role in the production of LPS-induced mediators. To determine whether PA participates in LPS-induced TLR4/NF-*κ*B pathway activation, we estimated the protein levels of TLR4, p-I*κ*B*α* and p-p65 in whole cell extracts, I*κ*B*α* in the cytoplasm, and p65 in the nucleus by western blot analysis. The research results show that LPS strongly stimulated TLR4, the phosphorylation of I*κ*B*α* and p65 in whole cell extracts and I*κ*B*α* degradation in the cytoplasm. It also caused the translocation of p65 into the nucleus. However, these effects were significantly inhibited by PA (**Figures 5A–G**). These results demonstrate that PA has protective effects against I*κ*Bα degradation and p65 translocation in LPS-treated IEC-6 cells.

### Role of TLR4/NF-κB Pathway on Intestinal Barrier Function, Cell Migration and NLRP3 Inflammasome in PA-Mediated IEC-6 Cells

**Figure 6:**
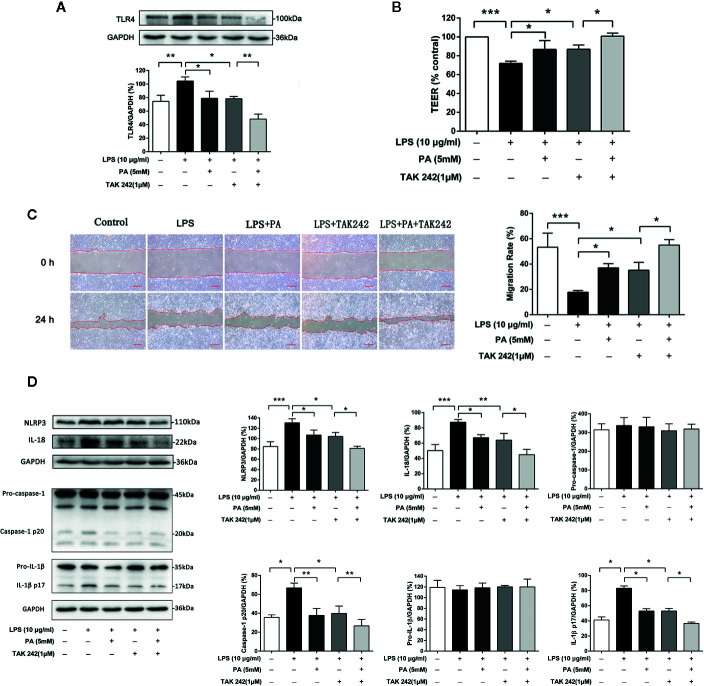
The effects of TLR4/NF-*κ*B pathway on intestinal barrier function, cell migration and NLRP3 inflammasome in IEC-6 cells. **(A)** The protein expression of TLR4 was determined by western blot analysis. **(B)** The value of TEER. **(C)** Images of cellular wounds generated were photographed at 0 and 24 h (scale bar: 0.3 mm). Wound healing of IEC-6 cells was assessed by comparing the wound area at 24 h after treatment with that prior to treatment. **(D)** The expressions of NLRP3, pro-caspase-1, caspase-1, pro-IL-1*β*, IL-1*β*, and IL-18 at protein levels were determined by western blot analysis. The data represent the averages ± S.D. Significant differences between the treatment and control groups are indicated as *P < 0.05, **P < 0.01, ***P < 0.001. Significance was determined by one-way ANOVA.

We also use TAK242, an inhibitor of TLR4, to further confirm whether TLR4/NF-*κ*B pathway was involved in PA-mediated cytoprotective effect. We found that treatment with TAK242 and PA further inhibited the expression of TLR4 in the IEC-6 cells (**Figure 6A**). As shown in **Figure 6B**, treatment with TAK242 and PA further elevated TEER. Meanwhile, as shown in **Figure 6C**, TAK242 increased the effect of PA on cell migration. Moreover, as shown in **Figure 6D**, TAK242 significantly decreased NLRP3 inflammasome activation which was inhibited by PA treatment. In summary, these results suggest that PA improves LPS-induced intestinal barrier function, increases cell migration and inhibits NLRP3 inflammasome *via* inhibition of the TLR4/NF-*κ*B pathway.

### The Protective Effects of PA on Ileal Tissues of Rats With LPS Injury

**Figure 7:**
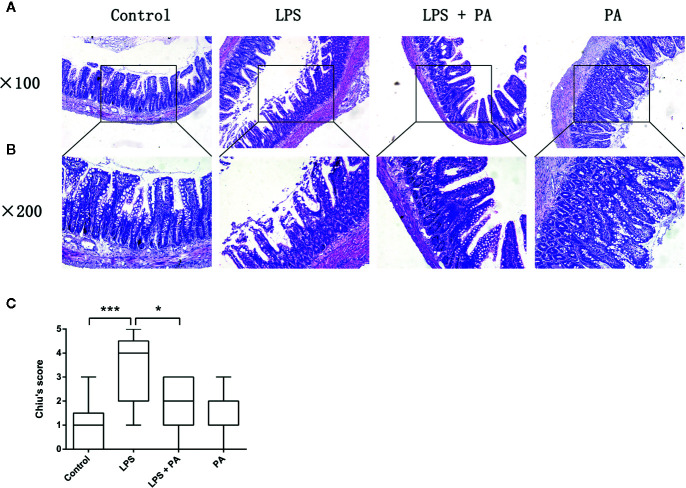
The effects of PA on intestinal histology. Histological sections of the ileum harvested 24 h after LPS treatment in rats with and without PA treatment. The ileal specimens were stained with hematoxylin and eosin and observed under a microscope of **(A)** 100 and **(B)** 200 magnifications. **(C)** Chiu’s score of intestinal specimens in each group. The data represent the averages ± S.D. Significant differences between the treatment and control groups are indicated as *P < 0.05, ***P < 0.001. Significance was determined by one-way ANOVA.

We evaluated the histology of ileal specimens stained with hematoxylin and eosin to determine the effects of PA on intestinal injury of rats with LPS injury (**Figures 7A, B**). Histopathologic evaluation of stained tissue sections showed no apparent alteration in the intestinal structure of control group and PA-treated rats. The ileal tissues from LPS-treated rats showed impaired intestinal villi, severe inflammatory cell infiltration, and locally necrotic areas. Compared with the LPS group, PA treatment to LPS-treated rats demonstrated less inflammatory cell infiltrate and alleviated villus injury in the ileum. As shown in **Figure 7C**, Chiu’s grading scale was further used to verify the histologic damage to the intestinal mucosa. These results suggest that PA plays a key role in protective effects on ileal tissues of rats with LPS injury.

### The Protective Effects of PA on Intestinal Barrier Function of Rats With LPS Injury

**Figure 8:**
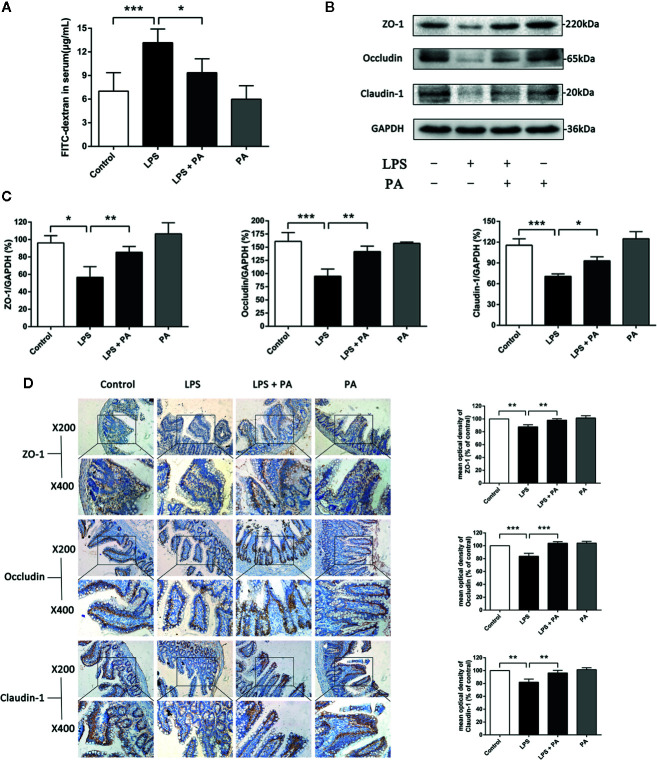
The effects of PA on intestinal permeability and tight junction protein expression in ileal tissues. *In vivo* intestinal permeability and tight junction protein expression in ileal tissues were assessed at 24 h after treatment of LPS. **(A)** The concentration of FD10S in the serum of rats. The higher level of FD10S in the serum indicates a higher intestinal permeability, resulting from a reduced barrier function of the intestine. **(B–C)** The protein expressions of ZO-1, occluding, and claudin-1 were determined by western blot analysis. **(D)** The protein expressions of ZO-1, occluding, and claudin-1 were determined by immunohistochemistry and observed under a microscope of 200 and 400 magnifications. The data represent the averages ± S.D. Significant differences between the treatment and control groups are indicated as *P < 0.05, **P < 0.01, ***P < 0.001. Significance was determined by one-way ANOVA.

The effects of PA on LPS-induced intestinal barrier dysfunction *in vivo* were subsequently explored. The concentration of fluorescent FD10S in the blood is an indicator of intestinal permeability *in vivo*. **Figure 8A** presented that rats in the LPS-treated group had higher levels of serum FD10S compared with the control group, indicating poorer barrier function (P < 0.001). Whereas rats’ blood in LPS + PA group had lower levels of FD10S compared with the LPS group (P < 0.05). We then assessed the expression of the tight junction proteins claudin-1, occluding, and ZO-1 by western blotting and immunohistochemistry. **Figures 8B–D** showed that the expression of tight junction proteins was decreased in rats of the LPS group. However, the expression levels of tight junction proteins were restored in the LPS-treated rats treated with PA. These findings indicate that treatment of PA can restore the intestinal barrier function of rats with LPS injury.

## Discussion

Intestinal epithelial cells have traditionally been considered passive cells responsible for maintaining the intestinal barrier function (Marchiando et al., 2010). In addition, intestinal inflammation has been associated with destruction of the intestinal barrier (Salim and Soderholm, 2011; Halpern and Denning, 2015). Clinically, anti-inflammatory drugs, steroids, and immunosuppressive drugs are traditionally used for the treatment of NEC or other intestinal inflammatory conditions (Suenaert et al., 2002; Cetin et al., 2004). However, the clinical application of these drugs is limited by their adverse effects, and there is therefore an urgent need to identify alternative remedies (Mao and Hu, 2016). PA, a final product of bacterial metabolism in the gut, is derived primarily from dietary fiber and has been reported to have extensive anti-inflammatory effects (Bolognini et al., 2016). Tong et al. found that PA could improve intestinal barrier function in DSS-induced mice (Tong et al., 2016). Tong et al. found that sodium propionate inhibited the down-regulation of tight junction proteins and improved the impaired intestinal barrier function induced by DSS. Moreover, sodium propionate inhibited oxidative stress in the colon. This was the first time PA has been shown to improve intestinal barrier function when used alone. However, this has been limited to PA acting on tight junction proteins to improve intestinal barrier function, and more mechanisms of PA acting on intestinal barrier function are to be explored. For instance, cell migration and NLRP3 inflammasome have been reported to be involved in intestinal barrier function (Reed et al., 2019; Zhuang et al., 2019). However, the protective effect of PA on the intestinal barrier function and the exact mechanism in LPS-induced intestinal barrier dysfunction are still unclear. In the current study, we investigated the role of PA in promoting the intestinal barrier function and its mechanism. We found that PA significantly improved intestinal barrier function by regulating cell migration and NLRP3 inflammasome through TLR4/NF-*κ*B signaling in LPS-induced IEC-6 cells. And, in *in vivo* study, it was shown that the intestinal barrier function of rats with LPS injury was restored by PA. Undeniably, there are some limitations in this paper, and it is also worth further study to explore how PA affects cell migration, NLRP3 inflammasome, and intestinal barrier function by acting on TLR4/NF-*κ*B signaling. The molecular association between cell migration and NLRP3 inflammasome of intestinal epithelial cells and intestinal barrier function is also worth further investigation.

Previous studies have shown that intestinal barrier injury is mainly manifested in increased permeability, and TEER and FD10S are the commonly used indicators of permeability (Zhang et al., 2016; Cremonini et al., 2017). Furthermore, Zonula occludens-1 (ZO-1), claudins, and occludin, as important components of tight junctions, play a role in limiting and regulating permeability (Almousa et al., 2018). In this study, claudin-1, occluding, and ZO-1 were selected as the representative proteins to study the effect of PA on the TJs. In addition, Chen et al. reported that the concentration of short-chain fatty acids (acetic, propionic and butyric acid) in the intestinal cavity was increased by feeding the mice with multiple fibers. Then, it was found that the expression of TJs was significantly increased and the intestinal barrier function was significantly changed (Chen et al., 2017). Elamin et al. also reported that pretreatment of Caco-2 cells with 4 mM PA significantly alleviated the ethanol-induced barrier dysfunction and TJs (Elamin et al., 2013). Consistent with previous studies, our results suggest that PA can significantly increase TJs, thereby effectively reducing intestinal barrier dysfunction *in vitro* and *in vivo* studies.

Various cellular mechanisms are involved in maintaining the intestinal barrier function, such as promoting cell migration and limiting NLRP3 inflammasome (Reed et al., 2019; Zhuang et al., 2019). It was found that intestinal epithelial cells can migrate quickly to maintain the intestinal barrier integrity when the intestinal epithelium is damaged by inflammation (Reed et al., 2019). However, the potential role of PA in cell migration has not been demonstrated. Thus, we used a scratch model to evaluate the effect of PA on cell migration. Our data showed that PA can alleviate the inhibition of LPS on cell migration. Intriguingly, we found treatment with PA alone also promoted cell migration without the stimulation of LPS. In previous studies, PA has been shown to be an energy source for intestinal epithelial cells (Cushing et al., 2015). Based on these findings, we consider that PA may play a role in promoting cell migration as an energy source for intestinal epithelial cells. However, whether PA provides energy for intestinal epithelial cells to promote cell migration needs further investigation. Futhermore, *β*1-integrin plays an important role in the dynamic regulation of epithelial cell adhesion and migration (Qureshi et al., 2005). Importantly, we found a decrease in the expression of *β*1-integrin treated with PA. In the current investigation, our results suggest that PA may promote cell migration by inhibiting the expression of β1-integrin in IEC-6 cells.

In a recent research, Feng et al. reported that inhibition of NLRP3 inflammasome could improve intestinal epithelial barrier function in Caco-2 cells (Feng et al., 2018). In order to investigate the effect of PA on the activation of NLRP3 inflammasome in LPS-induced IEC-6 cells, we examined the protein expression levels of NLRP3, caspase-1, IL-1*β*, and IL-18. Consistent with previous studies (Feng et al., 2018), our current results suggest that LPS significantly activates NLRP3 inflammasome. Conversely, PA treatment significantly attenuated activation of NLRP3 inflammasome. Taken together, our results suggest in some extent that PA may inhibit the activation of NLRP3 inflammasome to improve intestinal barrier function.

Intestinal epithelial cells express receptors extensively, including toll-like receptors (TLRs) (Kawai and Akira, 2010), of which TLR4 is the primary LPS receptor (Park and Lee, 2013). The TLR4/NF-*κ*B pathway has been identified as a major regulator of LPS-mediated inflammatory response. Once LPS binds to TLR4, the signaling cascade in the NF-*κ*B pathway is triggered, leading to the activation and subsequent induction of transcription factors (Zusso et al., 2019). However, the role of the TLR4/NF-*κ*B pathway in LPS-induced IEC-6 cells has not been reported. To further investigate the possible cellular mechanisms of PA in LPS-mediated IEC-6 cells, we measured the levels of TLR4 and phosphorylation of NF-*κ*B p65 in the cells. As expected, the levels of TLR4 and phosphorylation of NF-*κ*B p65 and translocation level of p65 into the nucleus were significantly reduced in the PA treatment group. This means that PA may inhibit the TLR4/NF-*κ*B signaling pathway to improve intestinal barrier function. To further explore the role of the TLR4/NF-*κ*B pathway in the protection of PA in LPS-induced IEC-6 cells, TLR4 inhibitor (TAK242) was used for further studies. The results showed that the PA combined with TAK242 significantly promoted cell migration, inhibited activation of NLRP3 inflammasome, and improved intestinal barrier function. There may be other signaling pathways involved in PA-induced intestinal barrier function. However, our current study suggests that the TLR4/NF-*κ*B pathway is at least partially involved in the protection of PA in LPS-induced intestinal barrier dysfunction.

**Figure 9:**
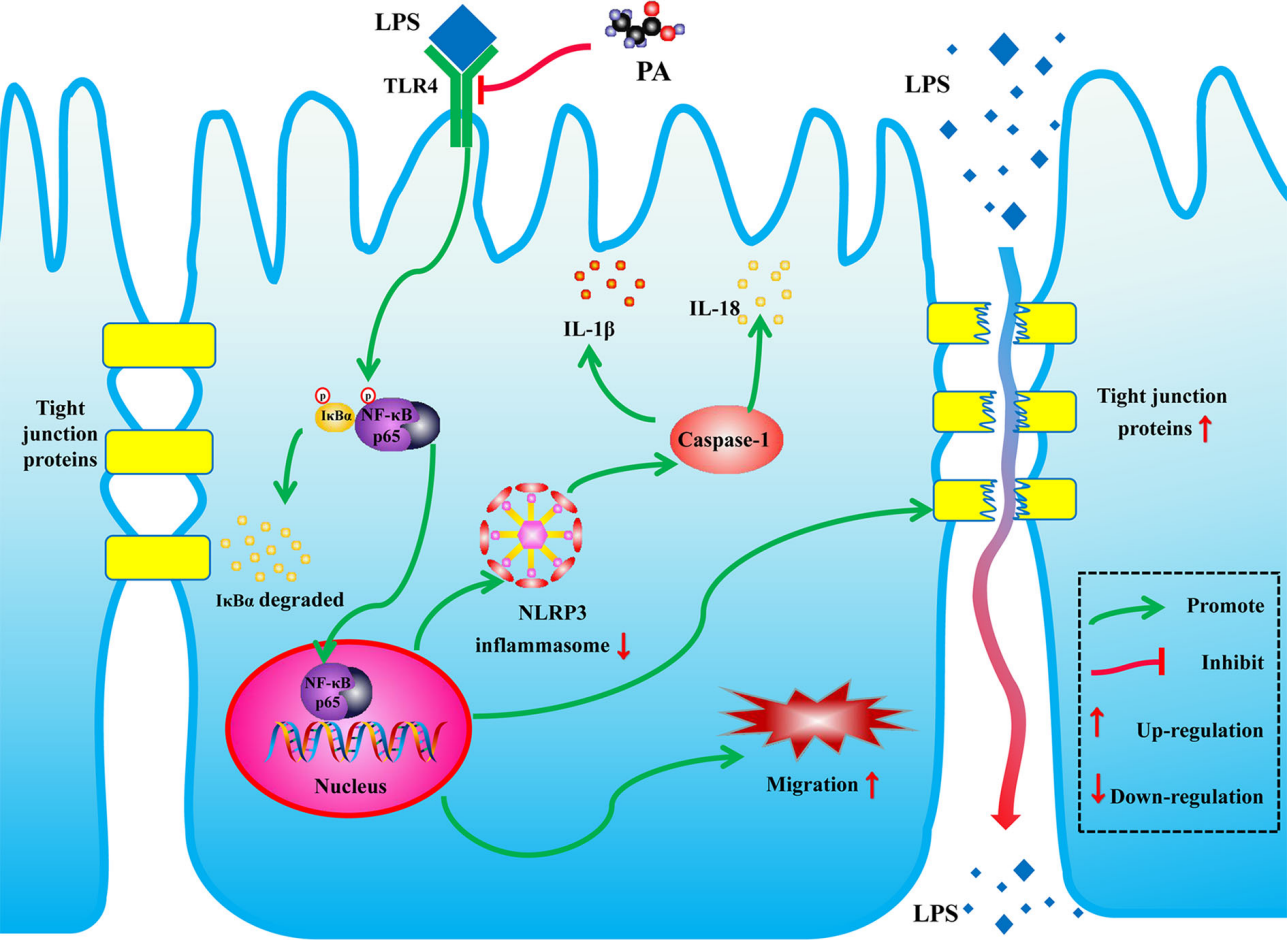
Schematic of the potential protective effects of PA in LPS-induced intestinal barrier dysfunction. PA is an effective substance that could prevent the LPS-induced intestinal barrier dysfunction *via* TLR4/NF-*κ*B pathway.

In conclusion, as shown in **Figure 9**, PA can promote cell migration, inhibit activation of NLRP3 inflammasome and thereby improve intestinal barrier function by inhibiting the TLR4/NF-*κ*B pathway. These findings reveal new potential therapeutic applications for PA in LPS-related intestinal barrier dysfunction.

## Data Availability Statement

The raw data supporting the conclusions of this article will be made available by the authors, without undue reservation, to any qualified researcher.

## Ethics Statement

The animal study was reviewed and approved by the Animal Care and Use Committee of Wenzhou Medical University.

## Author Contributions

Design of the study: ZL. Conduct of the study: RY, XH, XX, HC. Data collection: RY, XH, HF. Data analysis: RY, LZ. Manuscript writing: RY. All authors contributed to the article and approved the submitted version.

## Funding

This study is supported by Zhejiang Provincial Natural Science Foundation of China (LY17H040010), Zhejiang Province Medicine and Health Platform Project (2017ZD025) and Zhejiang Undergraduate Talent Project (2019R413079).

## Conflict of Interest

The authors declare that the research was conducted in the absence of any commercial or financial relationships that could be construed as a potential conflict of interest.
